# Human resources for health strategies adopted by providers in resource-limited settings to sustain long-term delivery of ART: a mixed-methods study from Uganda

**DOI:** 10.1186/s12960-016-0160-5

**Published:** 2016-10-19

**Authors:** Henry Zakumumpa, Modupe Oladunni Taiwo, Alex Muganzi, Freddie Ssengooba

**Affiliations:** 1 School of Public Health, Makerere University, Kampala, Uganda; 2 Department of Psychology, Obafemi Awolowo University, Osun State, Nigeria; 3 The Infectious Diseases Institute, Makerere University, Kampala, Uganda

**Keywords:** HIV, Health systems, Sustainability, Implementation science, Human resources for health

## Abstract

**Background:**

Human resources for health (HRH) constraints are a major barrier to the sustainability of antiretroviral therapy (ART) scale-up programs in Sub-Saharan Africa. Many prior approaches to HRH constraints have taken a top-down trend of generalized global strategies and policy guidelines. The objective of the study was to examine the human resources for health strategies adopted by front-line providers in Uganda to sustain ART delivery beyond the initial ART scale-up phase between 2004 and 2009.

**Methods:**

A two-phase mixed-methods approach was adopted. In the first phase, a survey of a nationally representative sample of health facilities (*n* = 195) across Uganda was conducted. The second phase involved in-depth interviews (*n =* 36) with ART clinic managers and staff of 6 of the 195 health facilities purposively selected from the first study phase. Quantitative data was analysed based on descriptive statistics, and qualitative data was analysed by coding and thematic analysis.

**Results:**

The identified strategies were categorized into five themes: (1) providing monetary and non-monetary incentives to health workers on busy ART clinic days; (2) workload reduction through spacing ART clinic appointments; (3) adopting training workshops in ART management as a motivation strategy for health workers; (4) adopting non-physician-centred staffing models; and (5) devising ART program leadership styles that enhanced health worker commitment.

**Conclusions:**

Facility-level strategies for responding to HRH constraints are feasible and can contribute to efforts to increase country ownership of HIV programs in resource-limited settings. Consideration of the human resources for health strategies identified in the study by ART program planners and managers could enhance the long-term sustainment of ART programs by providers in resource-limited settings.

## Background

Universal access to antiretroviral therapy (ART) is taking an increasing importance in global public health [1]. In 2014, UNAIDS released ambitious 90-90-90 global targets, a part of which aims at enrolling 90 % of those diagnosed with HIV on ART by 2020 [2]. The Sustainable Development Goals (SDGs) announced in September 2015 retained universal access to ART in the new international development agenda [3]. In November 2015, WHO issued new ART guidelines recommending that all diagnosed as HIV positive be enrolled on sustained ART regardless of disease stage [4].

Achieving these new global targets will necessitate overcoming a myriad of health system constraints in Sub-Saharan Africa which is the region with highest HIV burden in the world [5, 6]. These include sustaining funding to levels that are commensurate with the needs of the HIV response, securing ART commodity supply chains and resolving bottlenecks in service delivery [1, 7].

Within the health system building blocks, human resources for health (HRH) constitute a major barrier to the sustainability and further scale-up of ART in Sub-Saharan Africa [6–8]. From a total of 400,000 who were enrolled on ART in 2003 [9], the population enrolled on ART had risen to 15 million by mid-2015 [2].

This rapid expansion in ART coverage highlighted the human resources for health constraints associated with a dramatic expansion in patient volumes [10, 11]. ART scale-up occasioned heavy workloads on already over-burdened and demotivated health workers. HIV care and treatment has been consistently associated with health worker burnout [12–14].

Internal brain drain of health workers away from public and faith-based health facilities to NGO clinics where project-based salaries were more than twice as those in other health facilities, compounded the maldistribution of health workers [15–17]. Recent estimates suggest that health workforce vacancies in PEPFAR focus countries range from 50 to 79 % [18].

However, the human resources for health crisis in Sub-Saharan Africa is not only one of numbers. Health worker motivation challenges have characterized health service delivery for many years. This is demonstrated in the widespread absenteeism and low morale arising, partly, from unsatisfactory remuneration and negative work environments [19, 20].

Uganda is one of the 57 countries listed as having a human resources for health crisis and is consistently included in countries with acute shortages of health workers. Uganda has an estimated 1 health professional per 700 people which is below the WHO standard [21]. As is common in other countries in Sub-Saharan Africa, motivating the health workforce is a persistent challenge in Uganda. This is manifested in the widespread absenteeism and a high attrition rate of health workers [22–24].

Uganda has a high HIV burden and implemented an emergency national ART scale-up program between 2004 and 2009 with PEPFAR and Global Fund support [25]. ART scale-up commenced initially at national and regional referral hospitals with a gradual diffusion of these interventions to lower health facilities in a phased, decentralized service delivery approach [26]. ART scale-up was introduced on a trial basis and not as a long-term program [25]. The sustainment of these interventions has been a challenge for individual providers with human resources for health emerging as a key constraint.

Many previous responses to the human resources for health crisis in Sub-Saharan Africa have taken a top-down approach. These responses have been in a form of generalized global strategies and policy guidelines [27, 28]. There is a need expand the evidence base on bottom-up HRH strategies especially those devised by front-line providers in resource-limited settings. Moreover, there are mounting calls for locally derived HIV service delivery strategies drawn from the perspectives of providers in resource-limited settings [1, 5, 29].

The objective of the study was to examine the human resources for health strategies adopted at the facility level by providers in Uganda to sustain antiretroviral therapy (ART) beyond the ART scale-up phase between 2004 and 2009.

The study reported here is derived from a larger research project assessing the organizational and broader health system influences on the sustainability of ART programs.

## Methods

### Research design

The study adopted a mixed-methods approach involving both quantitative and qualitative data collection and analysis. The study was conducted sequentially in two phases [30]. The first study phase was conducted between January and April 2014. The second phase was conducted between August and October 2014.

#### Phase I: mixed-methods survey

In the first study phase, a survey of health facilities in Uganda was conducted to examine the human resources for health strategies adopted by providers to sustain ART service delivery since initial implementation of ART scale-up*.*

### Study sites and sample selection

A sample of 195 (out of 394) health facilities in Uganda which were accredited to provide ART between 2004 and 2009 was selected. Although a sample of 195 health facilities was targeted, a 75 % response rate was anticipated [31, 32]. We added 49 health facilities to our targeted sample of 195 health facilities to adjust for non-response [33]. Thus the final sample comprised of 244 health facilities.

Health facilities were selected through a two-stage process. Firstly, we secured the published Uganda Ministry of Health ART Monitoring Unit Report of March 2010 which lists all the 394 health facilities accredited to provide ART. The list of accredited ART providers in this report served as the sampling frame for the study. The 394 health facilities were placed in 10 strata based on their location in Uganda’s 10 geographic sub-regions as designated by The Uganda Bureau of Statistics. Using a lottery method, we then randomly sampled health facilities from each of the 10 strata based on proportionate representation. To ensure a nationally representative sample, we stratified by health facility type as shown in Table 1.

**Table 1:** Proportionate representation by health facility ownership

Health facility ownership	National sample (%)	Number of sites to sample
Public facilities	62	121
Private not for profit	18	35
Private for profit	17	33
Research and specialized clinics	3	6
Total	100	195

National sample based on the Ministry of Health March–June 2010 ART Monitoring Unit Report

A total of 227 (out of 244) health facilities returned filled questionnaires giving us a response rate of 93 %. A total of 195 questionnaires were retained for analysis.

### Data collection

During on-site visits, a hard copy questionnaire was handed to the head of the ART clinic of each of the 244 health facilities. The ART clinic managers were the respondents selected on the basis of their experience and knowledge of the ART program at their respective health facilities. A date and time was indicated when a research assistant would collect the filled questionnaire. To address potential non-response bias, phone call reminders were made to respondents every after 2 days [34]. The filled questionnaires were picked from respondents, on-site, a week after the initial visit.

### The study instrument

A mixed-methods questionnaire comprising close-ended and open-ended questions was used. The closed-ended questions sought to generate descriptive statistics relating to the number of health workers who served in the ART clinic compared to patient loads for each participating health facility and frequency distributions relating to the varied human resources for health strategies adopted by providers. The open-ended questions sought to explore the contexts and processes underpinning the HRH strategies adopted by health facilities to sustain long-term delivery of ART. A sample of the questions posed include 1. How did you ensure health workers remained motivated despite the increase in out-patient workloads? 2. What role did the leadership style of your ART clinic managers have on your commitment as a health worker?

The self-administered questionnaire was divided into three sections. The first section captured data on Health Facility characteristics such as facility type (public/private), location (rural/urban), year ART delivery was commenced, the range of HIV care and treatment services offered, and respondent demographics (sex, age, work experience).

The second section sought to capture data on staffing strength and patient load trends at baseline (March 2010) and at the time of data collection (April 2014). This section also elicited data on the staffing models adopted by providers such as the cadre of staff engaged in ART delivery (e.g. physician/non-physician-centred models).

The third section sought to elicit the human resource management strategies adopted by health facilities to sustain ART delivery since the initial implementation phase of 2004–2009 such as whether expert clients were used for filling staff shortages or if a ‘program champion’ was present at health facilities.

The questionnaire was pilot-tested among 12 ART clinic heads of health facilities outside the study sample to evaluate clarity and validity.

### Data analysis

Quantitative data from the self-administered questionnaire with respect to the close-ended responses relating to the varied human resource strategies adopted by providers was entered into Epi Data software (Version 3.1) and later exported into STATA software (Version 12.0). Descriptive statistics were generated and used to perform percentages, frequency counts and binary analyses.

#### Phase II: qualitative interviews

In the second study phase, we sought to gain an in-depth insight into the contexts underpinning the HRH strategies adopted at the facility level from the perspective of ART providers in Uganda.

The second phase involved in-depth interviews (*n =* 36) with individual health workers from 6 of the 195 health facilities purposively selected to represent ownership type (public/private), local setting (rural/urban), the different levels of care of the Ugandan health system (clinic, Health Centre III (sub-county level), Health Centre IV (sub-district), district hospital, regional referral hospital and national referral hospital [25, 26].

The selected health workers (*n =* 24) were those with leadership positions within the ART clinics (head of the ART clinic, head nurse, head of clinical services, human resource manager). In addition, two of the longest serving clinicians at each of the six health facilities (*n =* 12) were selected to elicit their perspectives on the HRH strategies adopted such as the influence of ART program leadership styles on health worker commitment.

An interview guide was constructed for the subsequent semi-structured interviews in the second phase of study for a more in-depth understanding of findings from the first study phase and to explore further the contexts and processes underpinning the HRH strategies adopted by providers in the surveyed health facilities.

### Data analysis

Qualitative data were analysed by way of coding and thematic analysis following the data analysis techniques described by Miles and Huberman [35]. Qualitative data were analysed for the two study phases. We sought to explore facility-level approaches to human resources for health constraints from the perspective of providers. To this end, responses to the open-ended items in the questionnaire formed the basis for devising the initial coding scheme.

For conceptual guidance, we adopted the definition of human resource management as that ‘concerned with the recruitment, selection, learning and development, reward, communication, teamwork and performance management’ [36]. In this study, we understood the term long-term sustainment to refer to continued delivery of antiretroviral therapy (ART) by a health facility since Uganda’s initial ART scale-up phase between 2004 and 2009. Sustainability was defined as ‘the continuation of programs and practices that were implemented within organizations, systems, or communities after initial implementation efforts or funding ended’ [37].

There were five emergent themes of HRH strategies adopted by providers based on qualitative analyses of the survey results following a team-based process that resolved discrepancies in coding and analysis.

In the second phase of the study, an in-depth exploration of the five emergent themes was done. The in-depth interviews with ART clinic managers were audiotaped and transcribed verbatim by two authors. The transcripts were read several times for familiarization. Codes were generated from the transcripts by two authors as guided by the predefined coding scheme from the first phase of the study. Codes which were not adequately captured by the initial coding scheme were collapsed under the five thematic strategies described in the findings.

Respondent validation interviews were conducted with five very experienced ART clinic managers to ensure accuracy in the assignment of codes and themes and the overall interpretation of the study findings [38].

Overall, five broad categories of human resources for health strategies adopted by providers for sustained delivery of ART emerged in the final analyses following a consensus process involving all the authors.

### Mixed-methods integration

Both qualitative and quantitative data were integrated following data analysis procedures suggested for mixed-methods sequential explanatory designs [30, 39, 40]. The themes that emerged from the qualitative data took priority in the analysis process owing to the study objective of identifying HRH strategies from the perspective of providers [30, 39–42]. Descriptive statistics were used for expansion of these emergent themes and for triangulation of data sources [30].

## Results

### Health facility characteristics

The study was conducted at 195 health facilities across Uganda. Nearly half of the health facilities (88 (45 %)) were located in peri-urban areas, compared to 76 (39 %) which were in urban areas and 27 (14 %) which were based in rural areas of Uganda.

Of the 195 health facilities; 121 were public facilities, 35 were private not for profit, 33 were private for profit and 6 were HIV research clinics.

**Fig. 1:**
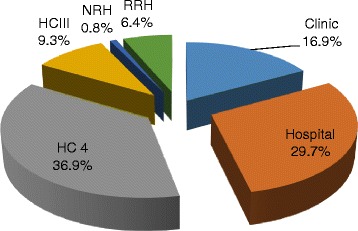
Representation by health facility size

In terms of facility size, Health Centre IVs were the most represented 72 (37 %), followed by hospitals at 58 (30 %), clinics at 33 (17 %) and regional referral hospitals at 12 (9.3 %) (Fig. 1).

Health facilities varied in when they started offering antiretroviral therapy (ART) with 21 % reporting that it was in 2004. Almost a half (47 %) reported this to have been between 2005 and 2006, followed by 21 % who indicated between 2007 and 2008 and 10 % in 2009.

### Characteristics of respondents

The study had a total of 236 respondents of whom 53 % were male (*n =* 125) and 47 % were female (*n =* 111). Most of the participants were in the age range of 41–45, and the overall mean work experience of respondents was 8 years (1–20).

In terms of cadre of staff, clinical officers at 77 (33.9 %) were the most represented, followed by nurses at 68 (30 %) and medical doctors at 55 (22.5 %).

#### Human resources for health strategies adopted

The results are presented in five sub-sections representing the five broad categorizations of the human resources for health strategies that emerged in the final analyses: (1) monetary and non-monetary incentives to health workers on busy ART clinic days; (2) workload reduction through spacing ART clinic appointments; (3) adopting training workshops as a motivation strategy for the workforce; (4) adopting non-physician-centred staffing models; and (5) devising program leadership styles that motivated the workforce in ART clinics.

#### Incentives to health workers on ART clinic days

Our findings show that providers adopted varied strategies to enhance the morale of health workers on days of the week designated as ART clinic days. The number of ART clinic days varied by provider but on average ranged between 1 and 3 days of the week. ART clinic days were described as congested and characterized by high out-patient workloads.

The workload at the clinic is high. Each clinician here handles about 50 clients a day. As a human being you need incentives. In fact because of these challenges some of our staff are ever absenting themselves. Like, I have four laboratory staff but on any given day there are only two on the ground. IDI 1107

Interviews with ART clinic managers revealed that a range of monetary and non-monetary incentives were devised to reduce staff absenteeism on these high patient volume days of the week. A range of non-monetary incentives were cited by a section of providers including the provision of soft drinks and meals served to health workers on ART clinic days.

We provide our staff with lunch and refreshments on ART Clinic days as an incentive to encourage them cope with the long patient lines. At times we give these refreshments in form of a lunch allowance paid out in cash instead of providing the actual soft drinks or meals. IDI 1115

In a network of six urban clinics participating in the study, participants reported that a salary top-up allowance was paid out monthly to health workers based on their attendance on ART clinic days. The funding was reported to be derived from a donor-funded HIV treatment capacity building project. Health facilities hosting HIV research initiatives, especially those involving Western universities, reported leveraging staff monetary allowances from the research grants associated with hosting them.

The Clinic attracts local and international researchers who bring in research grant funding which is then leveraged for the clinic through overheads and allowances for our staff in the clinic. IDI 1101

A range of relatively low-cost interventions by providers as in-house approaches aimed at reducing staff absenteeism and enhancing health workforce commitment on days of the week when workloads were especially high emerged strongly in the results.

The introduction of incentive schemes targeting health workers who worked in the ART clinics however created tension within health workers in some health facilities. In one of the public facilities, the in-charge reported that two core staff had been assigned to the ART clinic and despite the high patient volumes, the rest of the staff declined to relieve the burden on their colleagues because there were no monetary incentives for serving in the ART clinic as was the practice in neighbouring health facilities.

Staff here declined to come in and relieve the load of their colleagues in the ART clinic because they think this comes with monetary allowances. They think we receive this money and just don’t pass it on to them*.* IDI 1106

The results suggest that incentive schemes targeting select staff within the same health facility are not entirely seamless. They may result in tension and friction among health workers in some health facilities if considerations of fairness and equity are not taken into account.

#### Workload reduction through spacing clinic appointments

Interviews with providers revealed that ART scale-up stretched their staffing capacities and that they were overwhelmed by the rapidly expanded patient volumes.

In response to the increasing patient volumes and burgeoning workloads, providers reported varied workload reduction strategies to mitigate health worker burn out and to optimize clinician time. The approaches cited include adaptations to Uganda’s national ART guidelines by lengthening patient review periods from the recommended 3 to 6 months for patients deemed to be stable.

We lengthened the appointment periods for stable patients to ensure that only those patients who really deserved our clinician time came to the clinic. This has tremendously reduced the workload of our staff and decongested the clinic. IDI 1101

The national ART guidelines require that we review patients every 3 months. We introduced a refill program where stable patients are given longer review periods and only come in to pick drugs (ARVs). IDI 1202

The workload reduction strategies adopted by providers centred on spacing ART clinic appointments for patients. These measures were perceived to be effective in reducing health worker workload and burn out.

Table 2 shows that between March 2010 and April 2014, the number of health facilities with fewer than five staff reduced by 58 % (105 to 44). Within the same period, the number of health facilities with over 20 staff nearly tripled during the same period (10–29). Typically, the latter were health facilities at a higher level of care (such as referral hospitals) compared to the former which were lower-level health centres. A recent study in Uganda found that people living with HIV tended to bypass nearer ART sites and sought care in higher-tiered ART sites [43]. Workload reduction strategies such as through spacing ART clinic appointments and pharmacy-only refill programs are especially critical for higher-tiered ART sites with high patient loads.

**Table 2:** ART clinic staffing strength trends at participating health facilities

No. of staff	March 2010		April 2014		Change (2010 to 2014)	
	Number	%	Number	%	Number	%
1 to 5	105	54	44	23	−61	−58.1
6 to 10	57	29	56	29	−1	−1.8
11 to 15	13	7	43	22	30	230.8
16 to 20	10	5	23	12	13	130.0
20+	10	5	29	15	19	190.0
	195	100	195	100	0	0

#### ART training workshops as motivation

The findings show that training workshops and seminars in ART management were an important source of motivation for health workers at participating health facilities. The trainings were reported to be in the format of workshops and seminars conducted as short courses lasting a few days on-site but frequently off-site. The training workshops were said to be conducted on a regular basis by donor-funded intermediary agencies and Uganda’s Ministry of Health. Off-site training workshops were especially favoured by health workers because they were reported to attract monetary per-diem allowances or generous transport refunds. This was regarded as a form of supplemental income to health workers who widely perceived their remuneration as inadequate.

We don’t pay them that much and when there is a workshop we try to make sure that everyone gets a chance to go to one because it comes with money. We have a list. Who hasn’t gone for a workshop? They get the per-diem allowance and it makes them happy. IDI 1302

Providers reported that regular refresher trainings were a major facilitator of the strategy of task shifting of clinical tasks to non-physician cadre and enabled them to keep abreast with the continuous updates on ART management especially with regard to patient enrolment eligibility criteria and recommended ART regimens.

The heads of the ART clinic in some of the participating facilities revealed that they adopted in-service training in ART management as a recruitment strategy for new staff sourced from other sections of the health facilities to join the ART clinic.

When we need to recruit more staff for the ART clinic from other sections of the hospital, we ask for staff to be seconded to us. We then train these staff in ART after which they become part of us because they now have the skills and competencies we need in the ART clinic. IDI 1201

Qualitative analyses facilitated the characterization of health worker training in ART management as serving multiple human resource management functions of recruitment, retention and motivation.

#### Non-physician-centred staffing models

The results show that 93 % (181) of the 195 participating health facilities reported that non-physicians were involved in the clinical management of antiretroviral therapy (ART). Providers reported task shifting of clinical tasks from doctors to mid-cadre or staff with shorter training cycles at tertiary non-university institutions. The most cited mid-cadre categories were clinical officers, nurses and midwives who were reported to be the mainstay of antiretroviral therapy delivery in the participating health facilities. Community health workers (CHWs) and long-standing antiretroviral (ARV) users were reported to have been co-opted into ART management roles in a handful of providers. Health facilities which reported task shifting to non-physician cadre were more likely to perceive their ART programs as ‘very permanent’ compared to those where task shifting was not practised (*P* > 0.05).

A section of providers 36 (18 %) reported adopting a deliberate nurse-led ART service delivery model. Nurses were trained and empowered to perform multiple tasks within the HIV care and treatment continuum beyond their traditional scope of practice.

I think nurses are the best asset I have here. They will retrieve patient files. They will manage the reception. They will also dispense. HIV has brought out the potential of non-physician cadre. We used to think ‘oh those are just nurses’ but they can do a lot. And I think that’s our strength here. IDI 1201

Interviews with providers revealed a patient workload rationalization strategy in which nurses handled stable patients while more complicated cases were handled by medical doctors or senior clinicians on ART clinic days. This was reported to optimize clinician time for handling more complicated cases while leaving routine clinical management to mid-cadre.

The nurses help us to handle the heavy work load. They handle all our stable patients because patients are supposed to see a doctor at least once or a twice a year. IDI 1202

Task shifting to mid-cadre was perceived to have been aided by the standardization of ART delivery through national guidelines and the generation of simplified in-house manuals for mid-cadre in select providers.

##### The role of ‘expert clients’ in filling staffing gaps

A significant percentage of providers 59 % (115) reported relying on selected long-term ARV users known as ‘expert clients’ to relieve over-burdened staff by temporarily filling staffing vacancies at their sites especially on ART clinic days when they performed non-clinical tasks such as patient queue management, ARV drug pill counting and peer adherence counselling.

We selected some patients who help us with managing the large patient numbers. They help us in pill counting and repackaging of ARV drugs. They help us with the queues and they help us sort out those who are very sick with the help of nurses. They also do health education and share testimonies which help other patients in treatment adherence. They also do general counselling of their peers. IDI 1103

The findings suggest that providers responded to the shortage of physician-level cadre by expanding the traditional scope of practice of mid-cadre through role re-assignment. This strategy was facilitated by simplification of ART guidelines through in-house manuals as well as regular trainings in ART management.

#### Program leadership style as motivation

The results suggest that ART program leadership styles are influential on health workforce commitment. We found evidence that ART program staff responded positively to a leadership style where the ART clinic head did not supervise them too closely and described it as a source of motivation and a persistence enhancer in coping with heavy workloads in the context of rapidly expanded patient loads.

The Clinic Manager is not always on our case which makes us happy. We work on many patients but still come back the next day. IDI 1103

The head of the ART Clinic doesn’t call you asking where you are all the time. The Clinic head is a flexible leader and knows that we are all responsible adults. Although we may not be at the site at a particular time, say if we have over stayed the lunch break, we shall return and do what was expected of us during our absence. IDI 1302

During the qualitative interviews, ART clinic heads reported adopting non-financial incentives such as positive affirmation and constant recognition of health workers as an effective motivation strategy

As the Clinic Manager I have a habit of saying ‘Thank you’ for working. Whether they have worked or not. But I know it makes them feel appreciated. IDI 1203

##### The role of an internal program champion in health worker motivation

The presence of an internal program champion was associated with the motivation of health workers among staff we interviewed for the study. The program champion, typically the program leader, was described as an individual within the implementing organization who was strategically placed to foster ART program continuation by advocating for the needs of the program. The program champion was reported to be a figure within the organization who was instrumental in providing leadership and overall direction to the workforce in a manner that enhanced their long-term commitment to serve in the ART clinic. A program champion was characterized as an advocate within the organization who was key in resource mobilization for the maintenance of ART program activities from within and outside the organization.

The clinic manager single-handedly looks for grants and actively looks for new partners to support the clinic. The manager fights for the interests of the ART clinic within the central hospital administration. IDI 1203

Of the 195 participating health facilities, 53 % (103) reported the presence of a staff member within the implementing organization they regarded as a ‘program champion’.

The analysis suggests that ART program leadership styles and having an internal program champion were influential on health workforce commitment to ART service delivery at the participating health facilities.

## Discussion

Many previous responses to human resources for health (HRH) constraints have taken a top-down approach by way of generalized global strategies and policy guidelines. This study adopted a bottom-up approach by examining the HRH strategies devised by front-line providers in Uganda to sustain ART service delivery beyond the initial ART scale-up phase between 2004 and 2009. This study adds to the evidence base on measures by providers in resource-limited settings to motivate and retain the health workers in the face of rapidly expanded ART patient volumes. We sought a nationally representative sample of health facilities in Uganda which were accredited to provide ART between 2004 and 2009. The strategies identified were categorized into five themes.

Our findings demonstrate that providers devised a mix of monetary and non-monetary incentives to enhance the morale of health workers on ART clinic days which were characterized by high out-patient workloads. The results show that health workers responded positively to monetary incentives such as salary top-ups and lunch allowances on ART clinic days. ART program managers reported that monetary incentives enhanced the commitment of health workers and helped reduce absenteeism on busy ART clinic days. Our results do however highlight the need for caution in implementing vertical incentives for a segment of the workforce. At one of the participating health facilities, health workers declined to relieve the burden on the two core staff assigned to the ART clinic because there were no monetary incentives provided on ART clinic days as was the practice in neighbouring health facilities. This could be indicative of the potential of such incentives for creating tension among health workers not only within but also across health facilities. Our results also suggest that many of the health facilities derived monetary incentives from donor-funded projects which raises questions on the financial sustainability of such incentive schemes in resource-limited settings. The unintended or negative effects of selectively applied incentives to health workers especially those targeting ART scale-up have been noted [44, 45, 46].

Our findings also show that health workers responded positively to non-monetary incentives such as positive affirmation and program leadership styles that enhanced health worker commitment. The finding that non-monetary approaches are influential in sustaining health worker commitment is supported in the literature [47, 48]. In the context of resource-limited settings, non-monetary health worker incentives are especially appropriate. Training programs for ART clinic managers in adopting program leadership styles that enhance staff motivation are recommended. In this study, we found that 53 % of the health facilities reported the presence of an internal ‘program champion’ and this was a motivating factor for the health workforce. Identifying internal program champions in implementing organizations has been consistently associated with the sustainment of health care interventions [37, 49].

The majority of participating health facilities adopted non-physician-centred staffing models with the co-option of mid-cadre such as clinical officers, nurses and midwives in roles which, traditionally, were the preserve of physician cadre. A section of providers reported deliberately adopting a nurse-led care model. The study findings are consistent with previous studies which have associated HIV services scale-up success with task sharing of clinical tasks with non-physician cadre [9, 44, 50, 51].

In light of the extent of task shifting reported in our nationally representative sample and that this study adds to the accumulating evidence on task shifting*,* we recommend that non-physician cadre roles be formally expanded beyond ART delivery to include a wider range of programs including primary care services [51]. In the context of the rising non-communicable disease (NCD) epidemic in Sub-Saharan Africa, it is opportune to consider the potential role of task shifting approaches. It is common to find overflowing out-patient clinics for treating diabetes, cancers and cardiovascular disease in Uganda. With the requisite training and supervision, task shifting could ameliorate the HRH capacity constraints for managing the increasing caseload of chronic diseases in Uganda and other resource-limited settings. This study agrees with calls for the leveraging of ART scale-up lessons in the response to the NCD pandemic [52, 53].

The finding that 59 % of providers relied on select patients known us ‘expert clients’ for filling health worker shortages for non-clinical tasks such as peer counselling and queue management calls for further exploratory research on the use of ‘expert clients’ as a mainstream coping strategy for sustaining HIV service scale-up efforts. We call for an evaluation of their potential role in broader health service delivery goals such as realizing the topical universal health coverage (UHC) aspirations in resource-constrained settings. The involvement and role of ‘expert clients’ in HIV service delivery are consistent in the literature [54, 55].

### Alleviating HRH constraints through adaptations in ART service delivery models

Adaptations to current ART service delivery models are acknowledged as an important strategy for alleviating HRH constraints in resource-limited settings. Our results add to the literature documenting approaches to reducing workload through adaptions to traditional ART service delivery mechanisms. In this connection, previous studies have reported on community-supported models of care [56, 57]. These approaches have included ART appointment spacing, fast-track refills [58] and the use of community health workers (CHWs) to relieve the clinician workforce [59]. Differentiated care models where visits to clinics are based on assessment of individual patients rather than generalized guidelines have been called for [60]. Differentiated care models provide for reducing utilization of clinic-based care in favour of alternative care models which reduce the burden on the clinician workforce thereby motivating them [44]. Differentiated care models have been observed to be beneficial to patients by reducing costs associated with more frequent visits to the clinic and savings in transport and time [60, 61].

Modifications and adaptations to traditional ART service delivery models will be especially critical in resource-limited settings in the quest to meet the expansion in demand for ART arising from World Health Organization’s 2015 treatment guidelines which recommend that all diagnosed as HIV positive be enrolled on ART regardless of disease stage [60].

In our sample of health facilities, we observed variations in the implementation of integration of ART with other facility services. We found that vertical ART clinics are still common in Uganda and that several health facilities had health workers specifically assigned to ART clinics. Several studies have examined the pros and cons of vertical or disease-specific approaches [62–65]. There is evidence suggesting that vertical and integrated ART services can achieve similar outcomes [66]. As spelt out in WHO/UNAIDS’s Treatment 2.0 strategy, integration of ART with other services reduces the cost of health service delivery and is the future and long-term outlook for the sustainability of ART service delivery especially in resource-limited settings [1, 65, 66].

#### Limitations

This study had some limitations which we wish to acknowledge. We took a retrospective approach by selecting health facilities in Uganda which were accredited to provide ART between 2004 and 2009. We interviewed health facility managers who had been in service during this initial ART scale-up phase. Recall bias was a potential limitation given this approach. The study had some strengths as well. This study used a relatively large, nationally representative sample of 195 health facilities across Uganda which were accredited to provide ART between 2004 and 2009. The sample selected was broadly representative of ART service characteristics in Uganda during this period with regard to health facility size, location (rural vs. urban) and the diversity of the 10 geographic sub-regions of Uganda. The use of a mixed-methods approach was an additional strength of this study as it allowed us to move beyond the descriptive statistics generated such as the frequency distributions relating to the various health workforce strategies adopted, to an exploration of provider contexts and the processes involved in enhancing the motivation and retention of the available health workforce in a resource-constrained setting.

## Conclusions

Facility-level strategies for responding to human resources for health constraints are feasible and can contribute viable bottom-up solutions to efforts to increase country ownership of HIV programs in countries dependent on external donor support*.* ART clinic managers adopted varied monetary and non-monetary approaches to sustain health worker commitment in the context of rapidly expanded patient volumes and workloads at the participating health facilities in Uganda. Consideration of the human resources for health strategies identified in this study, by ART program planners, managers and funders could enhance the long-term sustainment of ART services by providers in resource-limited settings. Further research exploring the prospects of devising health worker motivation interventions targeting peak periods such as ART clinic days is recommended.
